# Microbiome Characterization of Infected Diabetic Foot Ulcers in Association With Clinical Outcomes: Traditional Cultures Versus Molecular Sequencing Methods

**DOI:** 10.3389/fcimb.2022.836699

**Published:** 2022-03-24

**Authors:** Hadar Mudrik-Zohar, Shaqed Carasso, Tal Gefen, Anat Zalmanovich, Michal Katzir, Yael Cohen, Yossi Paitan, Naama Geva-Zatorsky, Michal Chowers

**Affiliations:** ^1^ Department of Internal Medicine A, Meir Medical Center, Kfar Saba, Israel; ^2^ Sackler Faculty of Medicine, Tel-Aviv University, Tel-Aviv, Israel; ^3^ Rappaport Technion Integrated Cancer Center (TICC), Department of Cell Biology and Cancer Science, Rappaport Faculty of Medicine, The Technion – Israel Institute of Technology, Haifa, Israel; ^4^ Infectious Diseases Unit, Meir Medical Center, Kfar Saba, Israel; ^5^ Department of Orthopedics B, Meir Medical Center, Kfar Saba, Israel; ^6^ Clinical Microbiology Laboratory, Meir Medical Center, Kfar Saba, Israel; ^7^ Canadian Institute for Advanced Research (CIFAR), Toronto, ON, Canada

**Keywords:** diabetic foot ulcer, microbiome, 16S rRNA, metagenomics, amputation

## Abstract

**Background:**

Infected diabetic foot ulcers (IDFU) are a major complication of diabetes mellitus. These potentially limb-threatening ulcers are challenging to treat due to impaired wound healing characterizing diabetic patients and the complex microbial environment of these ulcers.

**Aim:**

To analyze the microbiome of IDFU in association with clinical outcomes.

**Methods:**

Wound biopsies from IDFU were obtained from hospitalized patients and were analyzed using traditional microbiology cultures, 16S rRNA sequencing and metagenomic sequencing. Patients’ characteristics, culture-based results and sequencing data were analyzed in association with clinical outcomes.

**Results:**

A total of 31 patients were enrolled. Gram-negative bacteria dominated the IDFU samples (79%, 59% and 54% of metagenomics, 16S rRNA and cultures results, respectively, p<0.001). 16S rRNA and metagenomic sequencing detected significantly more anaerobic bacteria, as compared to conventional cultures (59% and 76%, respectively vs. 26% in cultures, p=0.001). Culture-based results showed that *Staphylococcus aureus* was more prevalent among patients who were treated conservatively (p=0.048). In metagenomic analysis, the *Bacteroides* genus was more prevalent among patients who underwent amputation (p<0.001). Analysis of metagenomic-based functional data showed that antibiotic resistance genes and genes related to biofilm production and to bacterial virulent factors were more prevalent in IDFU that resulted in amputation (p<0.001).

**Conclusion:**

Sequencing tools uncover the complex biodiversity of IDFU and emphasize the high prevalence of anaerobes and Gram-negative bacteria in these ulcers. Furthermore, sequencing results highlight possible associations among certain genera, species, and bacterial functional genes to clinical outcomes.

## Introduction

Diabetic foot ulcers (DFU) develop in 25% of diabetic patients (Volmer Thole and Lobmann, 2016); half of which become infected (IDFU) and 68% cause osteomyelitis (Armstrong et al., 1998; Boulton et al., 2005; Lavery et al., 2006; Van Asten et al., 2016; Lavery et al., 2017). Approximately 12% of patients with IDFU require disabling lower limb amputation within five years of the initial foot lesion. These amputations cause functional deterioration and reduce quality of life (Armstrong et al., 1998; Volmer Thole and Lobmann, 2016).

The polymicrobial nature of the ulcers and the poor clinical response to long-term antibiotics, make IDFU challenging to treat (Hunt, 1992). Furthermore, it is complicated to differentiate culture-isolated causative pathogens from skin and environmental contaminants during acute exacerbation (Gardner et al., 2014). In some cases, it is not clear whether all isolated bacteria necessitate antibiotic treatment, especially when the sample is collected using superficial swabbing, rather than deep wound biopsies which are considered more reliable and therefore are recommended (Pellizzer et al., 2001; Treece et al., 2004; Lipsky et al., 2016; Lipsky et al., 2020). Lipsky et al. reported that patients with DFU with methicillin-resistant *Staphylococcus aureus (MRSA)* or *Pseudomonas* spp. Positive cultures exhibited good response to antibiotics that do not cover these organisms (Lipsky et al., 2005). This study also showed that *Pseudomonas aeruginosa* and *Enterococc*us spp. Colonized wounds without impairing wound healing (Lipsky et al., 2020). These findings reflect the limitations of cultures, that may reveal only partial results with respect to the diverse microbiota of the wounds (Dowd et al., 2008; Rhoads et al., 2012a; Rhoads et al., 2012b). Other well-known limitations of cultures are their selectivity to growth conditions and the risk of false-negative results due to prior antibiotic treatment (Kallstrom, 2014).

Improved diagnostic approaches are needed to decrease treatment failures, as the prevalence of diabetes mellitus is increasing worldwide, and consequently the number of patients dealing with diabetic foot ulcers is rising (Lavery et al., 2006). Molecular tools such as 16S rRNA and metagenomic sequencing provide detailed information about bacterial communities. However, data regarding the clinical implications of sequenced results from IDFU is scarce. Most published results to date focused on out-patients with uninfected ulcers and were limited in the taxonomic depth of analysis (Van Asten et al., 2016; Wolcott et al., 2016). Very few studies used metagenomic sequencing to obtain detailed mapping of the biodiversity of the ulcers, including functional genes (Suryaletha et al., 2018; Kalan et al., 2019). Here, we analyzed IDFU biopsies in hospitalized patients processed in parallel using cultures, 16S rRNA sequencing and metagenomics, and investigated microbial associations with clinical outcomes.

## Methods

### Study Setting and Population

The study was conducted April through September 2019 at Meir Medical Center (Kfar-Saba, Israel), a secondary medical center with 66,000 admissions per year, serving a population of 600,000 individuals. Inclusion criteria were: (a) age>18 years old (b) established diagnosis of type 2 diabetes mellitus (c) hospitalization to the Department of Orthopedics with IDFU during study period. Patients who had surgical intervention in the affected foot one month before enrollment or exhibited signs of another acute infection during hospitalization were excluded. Patients were divided into two groups according to clinical outcome: (a) “Conservative treatment” group (considered as the favorable clinical outcome) – included patients who were treated by intravenous antibiotic treatment with bedside debridement as needed, with no need of amputation up to 90 days post-discharge (b) “Amputation” group (considered as adverse clinical outcome) – included patients who underwent surgical toe resection or below knee amputation (BKA) during hospitalization, or within 90 days post-discharge. The clinical criteria for surgery were one of the following: (a) ulcers with extensive involvement of necrotizing soft tissue with poor response to IV antibiotic treatment (b) systemic inflammatory symptoms with no other source of infection and no response to IV antibiotic treatment. (c) chronic osteomyelitis of proximal phalanges, especially in cases with progressive bone destruction.

Antibiotic therapy during hospitalization was given at the discretion of the treating physician, and in accordance with available cultures.

### Data Collection

**Figure 1:**
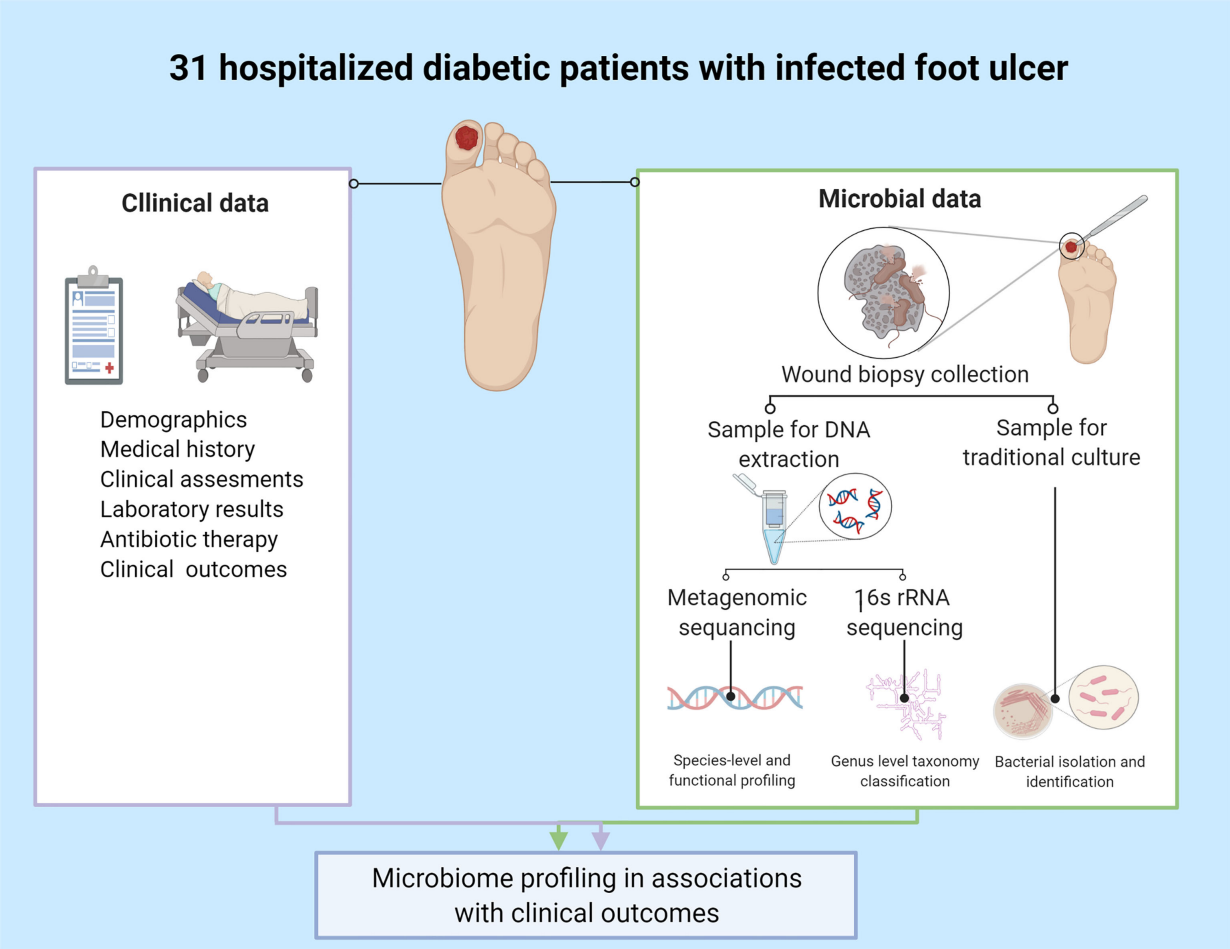
Study design. Created with BioRender.com.

Clinical data: Demographics, co-morbidities, laboratory measures, radiology imaging results and antibiotics prescribed one month before enrollment and given during hospitalization were collected from electronic medical records of all participants (**Figure 1**). The infected diabetic foot ulcer of each patient was clinically evaluated by an orthopedic surgeon or an infectious diseases physician upon admission. A uniform protocol was used, based on Texas and IWGDF/IDSA classifications (Lavery et al., 1996; Lipsky et al., 2012; Lipsky et al., 2016; Lipsky et al., 2020), which included ulcer’s location, size, clinical signs of cellulitis, abscess or osteomyelitis, systemic toxicity, vascular and neurologic status. Osteomyelitis was diagnosed using the well-known “probe-to-bone” clinical diagnostic tool, supported by X-ray findings.

Clinical outcomes measured: Length of stay (LOS), days of antibiotic therapy in-hospital, below knee or toe amputation (up to 90 days post discharge) and recurrent admissions.

Deep tissue biopsy collection: Biopsies were obtained by orthopedic surgeons after sterilization and debridement of superficial necrotic tissues. Two samples were collected from each participant: one for culture and the other for DNA extraction. The samples were collected simultaneously or no more than 48 hours apart.

Culture techniques: Biopsies were cultured in the microbiology Laboratory at Meir Medical Center. See **Supplementary Materials** for detailed culturing protocol.

DNA extraction and sequencing: Biopsies for DNA extraction were kept at -80°C within one hour after collection and were sent to the Geva-Zatorsky Laboratory, at the Technion Faculty of Medicine, Haifa, for extraction. Detailed extraction protocol is summarized in the **Supplementary Materials**. 16S rRNA and metagenomic sequencing were performed for each biopsy at the Sequencing Core at the University of Illinois at Chicago. For sequencing protocols, see **Supplementary Materials**.

### Statistics and Bioinformatics

Data were presented as number and percentage for nominal variables and as mean or median for continuous data. Categorical variables were compared using the chi-square test or Fisher-Exact test. Continuous data were compared using the T-test or the Mann-Whitney-U test. Friedman-test for non-parametric related samples was used to find differences among the three methods. P<0.05 was considered statistically significant. Data were analysed using SPSS, version 25 (IBM Corp., Armonk, NY).

Taxonomic data were filtered for bacteria and subsequently filtered by a minimum of 0.1% relative abundance and detection in at least 3 samples. Description of the analysis methods and details regarding the availability of the raw sequenced data are provided in the **Supplementary Materials**.

Two levels of analysis in association with clinical outcomes were performed:

(a) Comparison between culture and 16S rRNA sequencing results (N=30)

(b) Comparison between culture, 16S rRNA and metagenomic sequencing results (N=13).

### Ethics

The study was performed in accordance with the declaration of Helsinki and was approved by the Institutional Ethics Committee of Meir Medical Center (0143-18MMC). Patients provided signed informed consent.

## Results

### Patients’ Characteristics

During the six-month study period, a total of 62 wound samples were obtained from 31 patients who met the inclusion criteria. The study population characteristics are outlined in **Table 1**. Mean age was 62 years and 81% were men. Mean glycosylated hemoglobin was 8.8% and 94% were diagnosed with osteomyelitis. Twenty-three individuals underwent amputation (toe resection in 19 patients and BKA in 4) during hospitalization or within 90 days of discharge. There were no differences in co-morbidities, Charlson Comorbidity Index, LOS and recurrent admissions between patients who underwent amputation (N=23) and those who were treated conservatively (N=8, **Table 1**).

**Table 1:** Patient characteristics at enrolment and clinical outcomes measured.

Characteristic	Total (N=31)	Amputation (N=23)	Conservative treatment (N=8)	P-value
Age, years, mean (± SD)	62 ( ± 13.3)	61 ( ± 12.9)	64 ( ± 15.2)	0.65
Men, N (%)	25 (81)	18 (78)	7 (87)	0.50
Ulcer location				
Forefoot ulcers (number of toes involved**),** N (%)				0.34
1 toe	25 (81)	18 (78)	7 (87)	
>1 toe	5 (16)	4 (17)	1 (13)	
Hindfoot ulcers	1 (3)	1 (4)	0 (0)	
Ulcer size, N (%)				0.50
<3cm	25 (81)	18 (78)	7 (88)	
3-10cm	6 (19)	5 (22)	1 (13)	
Infection clinical evaluation, N (%)				
Cellulitis	13 (42)	11(48)	2 (25)	0.24
Osteomyelitis (OM)	29 (94)	21 (91)	8 (100)	0.54
Abscess	1 (3)	1 (4)	0 (0)	0.74
X-ray positive for OM, N (%)	21 (68)	15 (65)	6 (75)	0.48
Texas classification				
Stage B	17 (54)	12 (52)	5 (62)	0.70
Stage D	14 (45)	11(48)	3 (37)	
Grade 3 IWGDF/IDSA System classification	29 (93)	21 (91)	8 (100)	0.60
PEDIS 2	1	1	0	0.21
PEDIS 3	15	9	6	
PEDIS 4	15	13	2	
C-Reactive protein (mg/L) at admission, mean (± SD)	12 ( ± 9.3)	13 ( ± 8.9)	7 ( ± 9.5)	0.11
WBC (k/uL) at admission, mean (± SD)	12 ( ± 4.5)	13 ( ± 4.8)	10 ( ± 2.8)	0.14
Hemoglobin at admission (g/dL), mean (± SD)	11.3	11.04	11.38	0.77
Albumin at admission (g/dL), mean (± SD)	3.28	3.27	3.47	0.054
Antibiotic exposure within 30 days pre-admission, N (%)				
No exposure	19 (61)	13 (56)	6 (75)	0.43
Antibiotic prescriptions	12 (39)	10 (43)	2 (25)	0.57
Anaerobic coverage	9 (29)	7 (30)	2 (25)	
Previous toe amputation, N (%)	12 (39)	8 (35)	4 (50)	0.36
Co-morbidities, N (%)				
Essential hypertension	21 (68)	15 (65)	6 (75)	0.48
Peripheral vascular disease^a^	11 (35)	10 (43)	1 (12)	0.12
Neuropathy	30 (97)	23 (100)	7 (88)	0.80
Dyslipidemia	19 (61)	12 (52)	7 (88)	0.09
Congestive heart failure	9 (29)	6 (26)	3 (38)	0.42
Ischemic heart disease	8 (26)	6 (26)	2 (25)	0.67
Paroxysmal atrial fibrillation	4 (13)	3 (13)	1 (12)	0.73
Cerebrovascular disease	7 (23)	7 (30)	0 (0)	0.09
Chronic kidney disease	3 (10)	1 (4)	2 (25)	0.16
Chronic obstructive pulmonary disease	1 (3)	1 (4)	0 (0)	0.74
Smoking				
Smoker	7 (23)	5 (22)	2 (25)	0.45
Past smoker	3 (10)	2 (9)	1 (12)	0.45
Recent glycosylated hemoglobin, mean (± SD)	8.8 ( ± 2)	8.8 ( ± 2)	9.0 ( ± 2)	0.72
Charlson Comorbidity Index^b^ N (%)				0.86
0-3	6 (19)	5 (24)	1 (10)	
4-5	6 (19)	4 (17)	2 (25)	
>6	19 (61)	12 (52)	7 (88)	
Outcomes measured				
Hospitalization duration, mean (± SD)	14 ( ± 11)	15 ( ± 12)	10 ( ± 9)	0.31
Days of antibiotic therapy, mean (± SD)	13 ( ± 11)	13 ( ± 11)	14 ( ± 14)	0.54
Rec admission N (%)	8 (26)	6 (26)	2 (25)	0.95

a Peripheral vascular assessments are provided in **Table S1** in the **Supplementary Materials**.

b Charlson Comorbidity Index score: 0-3 = 77%-98% 10-year survival rate, 4-5 = 21-53% and >6 = 0-2%.

### Microbial Data Analysis

Biopsies were obtained within a median of 3 (IQR 1-4) days from admission. Antibiotic treatment was given for a median of one day (IQR 0-2) before biopsy collection. Bacteria were isolated in 30 culture samples (97%). All 31 DNA samples were sequenced successfully using 16S rRNA and metagenomic sequencing. Thirty 16S rRNA samples and 13 metagenomic samples were defined as high quality and included in the analysis.

**Figure 2:**
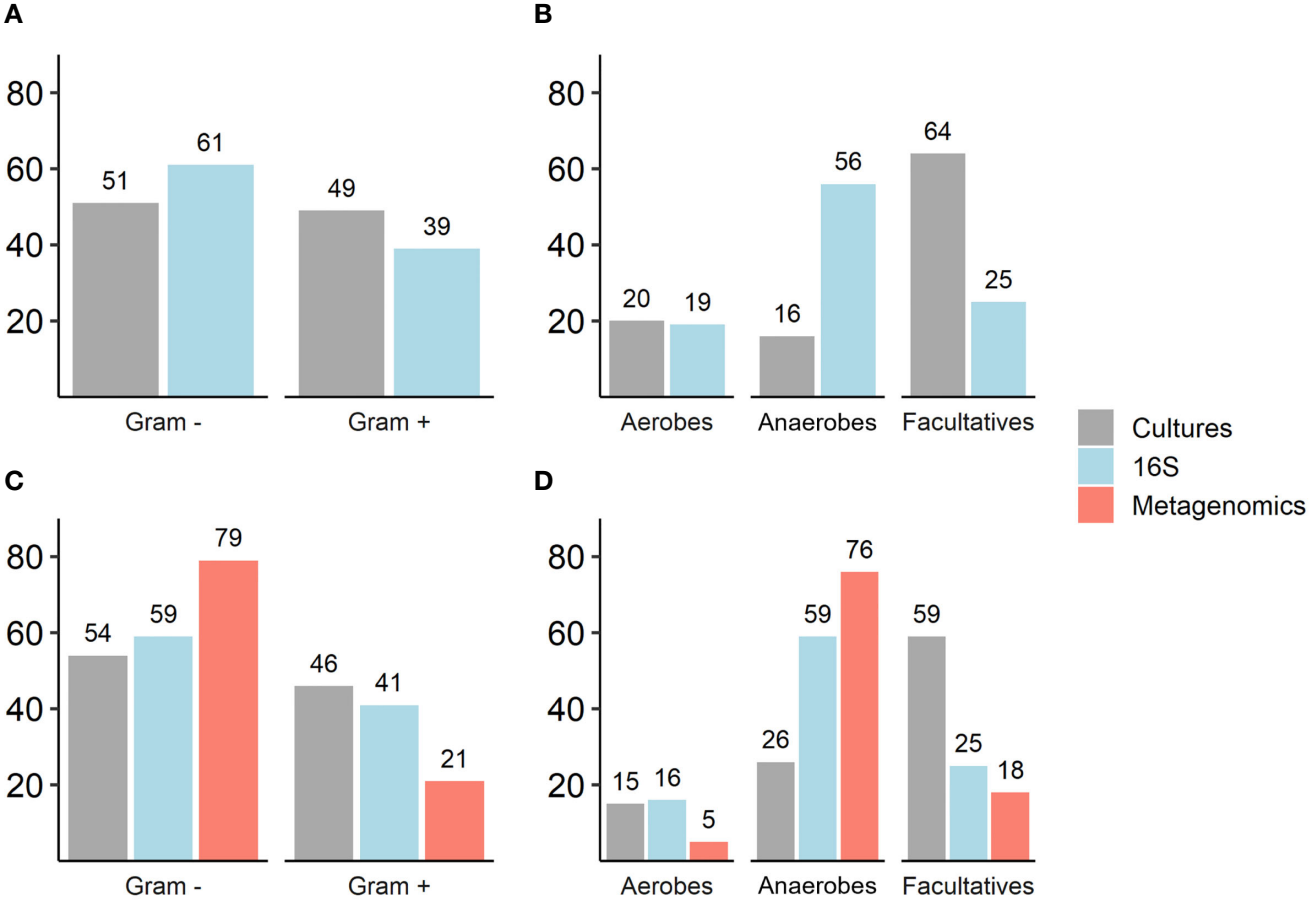
**(A–D)**. Comparison of Gram staining characteristics and oxygen requirements of isolated and sequenced bacteria as reflected by traditional cultures, 16S rRNA and metagenomic sequencing. Numbers represent percentages (%); **(A, B)** Traditional cultures vs. 16S rRNA sequencing results (N=30): Gram staining, p=0.07; oxygen requirements (anaerobes and facultative anaerobes, p<0.001). **(C, D)** Traditional cultures, 16S rRNA sequencing and metagenomic sequencing results (N=13): Gram staining, p=0.01; oxygen requirements (anaerobes, p<0.001; facultative anaerobes, p=0.001).

**Figure 3:**
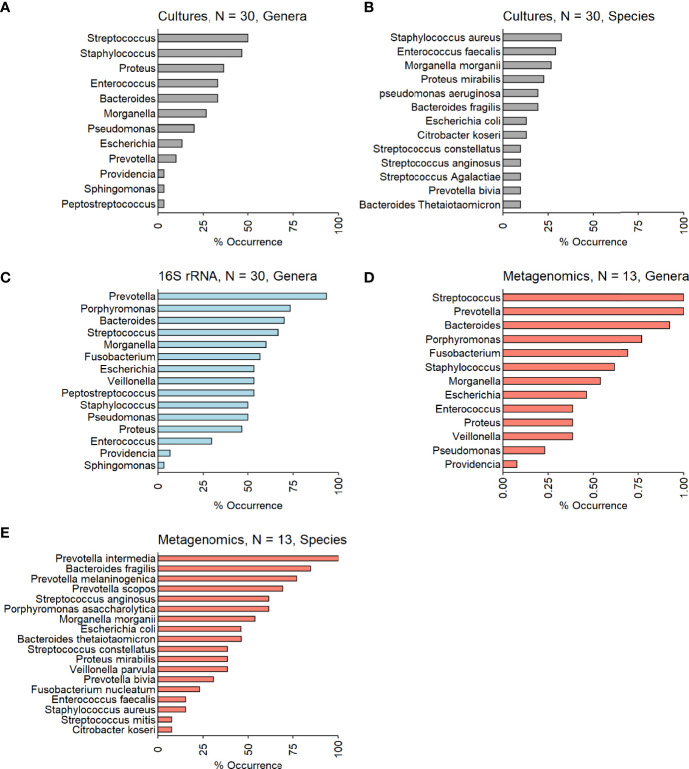
**(A–E)**. Occurrences of the most prevalent bacteria among the investigated IDFU. **(A, B)** Cultures results (genera and species level – N=30). **(C)** 16S rRNA sequencing results (genera level – N=30). **(D, E)** Metagenomic sequencing results (genera and species level – N=13).

Culture results: Thirty-two different bacteria were identified across patients with a mean of four bacteria per ulcer; there were no differences in mean bacterial number in culture-based results between patients who were conservatively treated and those who underwent amputation (**Table 2**). Gram-negative bacteria (GNB) and facultative anaerobes were the most prevalent (51% and 64%, respectively, **Figure 2A, B**). *Streptococcus*, *Staphylococcus*, *Proteus*, *Enterococcus* and *Bacteroides* were the five most dominant genera (**Figure 3A**), while *Staphylococcus aureus*, *Enterococcus fecalis*, *Morganella morganii*, *Proteus mirabilis* and *Pseudomonas aeruginosa* were the five most dominant species (**Figure 3B**). Notably, *Staphylococcus aureus* was more prevalent among patients who were treated conservatively (63% vs. 23%, p=0.048, **Table 2**). Bacteria with AMP-C β-lactamase resistance mechanism were more prevalent in patients who underwent amputation (39% vs. 0, p=0.04, **Table 2**). All other recognized resistance mechanisms were distributed similarly among the patients.

**Table 2:** Overview of traditional cultures, 16S rRNA sequencing and metagenomic sequencing results.

	Total (N=31)	Amputation (N=23)	Conservative treatment (N=8)	P-value
**Culture results**				
Patients with positive culture results, N (%)	30 (97)	22 (96)	8 (100)	0.79
Number of bacteria per patient, mean (± SD)	4.0 ( ± 1.8)	4.4 ( ± 2.7)	3.8 ( ± 1.5)	0.45
Gram stain, mean (± SD)				
Gram-positive bacteria	1.9 ( ± 1.2)	1.8 ( ± 1.1)	2.2 ( ± 1.6)	0.37
Gram-negative bacteria	2.2 ( ± 1.4)	2.3 ( ± 1.3)	1.9 ( ± 1.8)	0.52
Oxygen requirements, mean (± SD)				
Aerobic bacteria	0.6 ( ± 0.7)	0.7 ( ± 0.7)	0.5 ( ± 0.5)	0.48
Anaerobic bacteria	0.7 ( ± 0.9)	0.6 ( ± 0.6)	1.0 ( ± 1.4)	0.35
Facultative anaerobic bacteria	2.7 ( ± 1.6)	2.7 ( ± 1.6)	2.6 ( ± 1.8)	0.92
5 most prevalent species – occurrences, N (%)				
*Staphylococcus aureus*	10 (33)	5 (23)	5 (63)	0.048
*Enterococcus fecalis*	9 (30)	7 (32)	2 (25)	0.57
*Morganella morganii*	8 (26)	6 (27)	2(25)	0.14
*Proteus mirabilis*	7 (23)	3 (14)	4 (50)	0.053
*Pseudomonas aeruginosa*	6 (20)	5 (23)	1 (12)	0.50
Resistance mechanisms, N (%)				
Any resistance	13 (43)	11 (50)	2 (25)	0.24
AMP-C	9 (30)	9 (41)	0 (0)	0.041
ESBL	1 (3)	1 (4)	0 (0)	0.74
MRSA	3 (10)	2 (9)	1 (12)	0.60
Pseudomonas aeruginosa MDR	1 (3)	1 (4)	0 (0)	0.74
Carbapenem resistant Enterobacteriaceae	2 (7)	1 (4)	1 (12)	0.46
**16s rRNA sequencing results**				
Patients with high quality results, N (%)	30 (97)	22 (96)	8 (100)	0.79
Number of genera per patient^*^, mean (± SD)	26.9 ( ± 12.9)	28.5 ( ± 11.8)	22 ( ± 12)	0.19
Gram stain, mean (± SD)				
Gram-positive bacteria	10.7 ( ± 5.3)	11.4 ( ± 5.3)	8.5 ( ± 5.3)	0.20
Gram-negative bacteria	16.2 ( ± 7.4)	17.0 ( ± 7.5)	13.9 ( ± 7.1)	0.30
Oxygen requirements, mean (± SD)				
Aerobic bacteria	5.1 ( ± 5.0)	5.1 ( ± 5.0)	5.1 ( ± 5.0)	0.99
Anaerobic bacteria	15.1 ( ± 8.7)	16.4 ( ± 8.4)	11.4 ( ± 8.7)	0.18
Facultative anaerobic bacteria, mean	6.7 ( ± 3.2)	7.0 ( ± 3.1)	5.9 ( ± 3.1)	0.47
5 most prevalent genera – occurrences, N (%)				
*Prevotella*	28 (93)	21 (70)	7 (23)	
*Prophyromonas*	22 (73)	18 (60)	4 (13)	
*Bacteroides*	21 (70)	19 (63)	2 (7)	
*Streptococcus*	20 (67)	14 (47)	6 (20)	
*Morganella*	18 (60)	14 (47)	4 (13)	
**Metagenomics sequencing results**				
Patients with high quality results, N (%)	13 (42)	10 (43)	3 (37)	0.80
Number of species^a^, mean (± SD)	26.1 (7.9)	26.8 (7.6)	23.7 (10.0)	0.47
Gram stain, mean (± SD)				
Gram-positive bacteria	5.4 (3.1)	5.2 (3.5)	6.0 (1.7)	0.81
Gram-negative bacteria	20.6 (8.1)	21.5 (7.9)	17.7 (9.6)	0.47
Oxygen requirements, mean (± SD)				
Aerobic bacteria	1.5 (1.0)	1.5 (1.2)	1.3 (0.6)	0.94
Anaerobic bacteria	19.8 (6.8)	20.8 (6.2)	16.3 (9.0)	0.31
Facultative anaerobic bacteria	4.5 (2.4)	4.1 (2.1)	5.7 (3.2)	0.29
5 most prevalent species – occurrence, N (%)				
*Prevotella intermedia*	13 (100)	10 (77)	3 (23)	
*Bacteroides fragilis*	11 (85)	9 (69)	2 (16)	
*Prevotella melaninogenica*	10 (77)	8 (61)	2 (16)	
*Prevotella scopos*	9 (69)	7 (53)	2 (16)	
*Porphyromonas asaccharolytica*	8 (61)	7 (53)	1 (8)	

a Taxonomic data were subsequently filtered by a minimum of 0.1% relative abundance and detection in at least 3 samples.

16S rRNA sequencing results: A mean of 27 genera per sample were identified with predominance of anaerobes and GNB (56% and 61% respectively, **Figures 2A, B**). *Prevotella*, *Porphyromonas*, *Bacteroides*, *Streptococcus* and *Morganella* were the five most prevalent genera (**Figure 3C**). Mean relative abundances of the most abundant bacteria are demonstrated in **Figure S1A** in the **Supplementary Materials**. No associations between specific genera and clinical outcomes were found.

**Figure 4:**
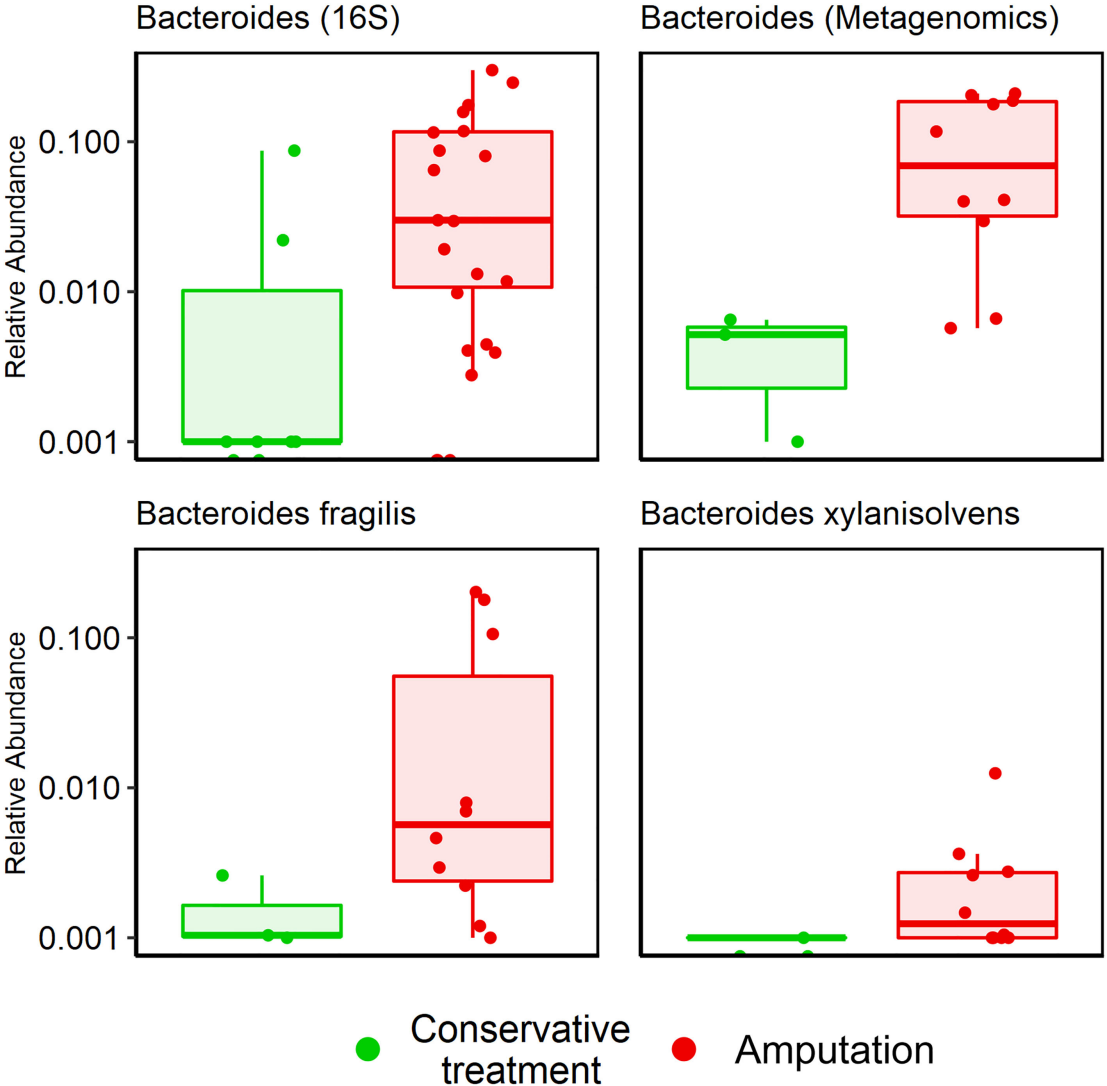
Association between *Bacteroides* genus and amputation according to 16S rRNA sequencing and metagenomic sequencing. Bacteroides genus, p<0.001*; Bacteroides fragilis*, p=0.04; *Bacteroides xylanisolvens*, p=0.002.

Metagenomic results: A mean of 26 species per sample were demonstrated (**Table 2**). GNB and anaerobes predominated (79% and 76% respectively, **Figures 2C, D**). The five most prevalent species were *Prevotella intermedia*, *Bacteroides fragilis*, *Prevotella melaninogenica*, *Prevotella scopos and Porphyromonas asaccharolytica* (**Figure 3E**). Mean relative abundances of the most abundant bacteria are demonstrated in **Figures S1B, C** in the **Supplementary Materials**. Bacteroides genus was more common among patients who underwent amputation compared with those who were treated conservatively (p<0.001, **Figure 4**). Species level analysis showed that *Bacteroides fragilis* and *Bacteroides xylanisolvens* predominated IDFU of patients who underwent amputation (p=0.04, p=0.002, respectively, **Figure 4**), while *Proteus mirabilis*, *Pseudomonas aeruginosa*, *Streptococcus agalactiae* and *Escherichia coli* were more prevalent among patients who were treated conservatively (p=0.02, p<0.001, p=0.002, p=0.04, respectively, **Figure S2**).

Comparison between investigative tools: 16S rRNA sequencing recognized 85% of the genera and metagenomic sequencing recognized all the species isolated in cultures. Overall, GNB were more prevalent than Gram-positive bacteria; this was most pronounced in the sequenced data. Anaerobes predominated in 16S rRNA and metagenomic sequencing compared to culture results (**Figure 2**).

**Figure 5:**
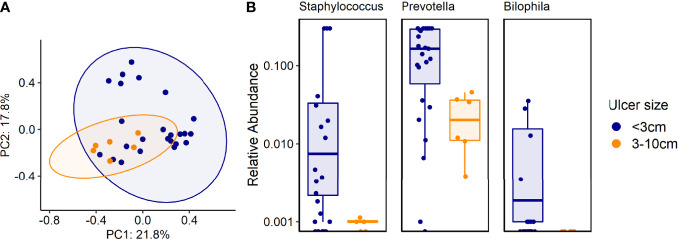
**(A, B)**. Associations between certain genera and species and ulcer size. **(A)** Beta diversity (Bray-Curtis dissimilarity index) of samples from patients with different ulcer sizes: PCoA plot of taxonomic features shows the clustering of samples according to ulcer sizes. Ellipsoids represent a 95% confidence interval surrounding each group. p<0.01 (PERMANOVA). **(B)** 16S rRNA results analysis: Shown are genera that were more common in ulcers smaller than 3cm, compared with ulcers 3-10cm, p<0.001.

Ulcer size: Culture results did not reveal any significant association between specific genera or species and ulcer size. However, certain bacteria correlated with ulcer size in sequenced data results; *Staphylococcus*, *Prevotella* and *Bilophila* genera were more common in ulcers <3 cm, compared with ulcers 3-10 cm, according to 16S rRNA sequencing (p<0.001, **Figure 5**). Metagenomics provided more in-depth characterization, revealing 12 species that were more prevalent in small ulcers and 21 species in larger ulcers (**Table S3** and **Figures S3, S4**). We did not find correlations between ulcer size and clinical outcomes (**Table 1**).

### Functional Data Analysis

**Figure 6:**
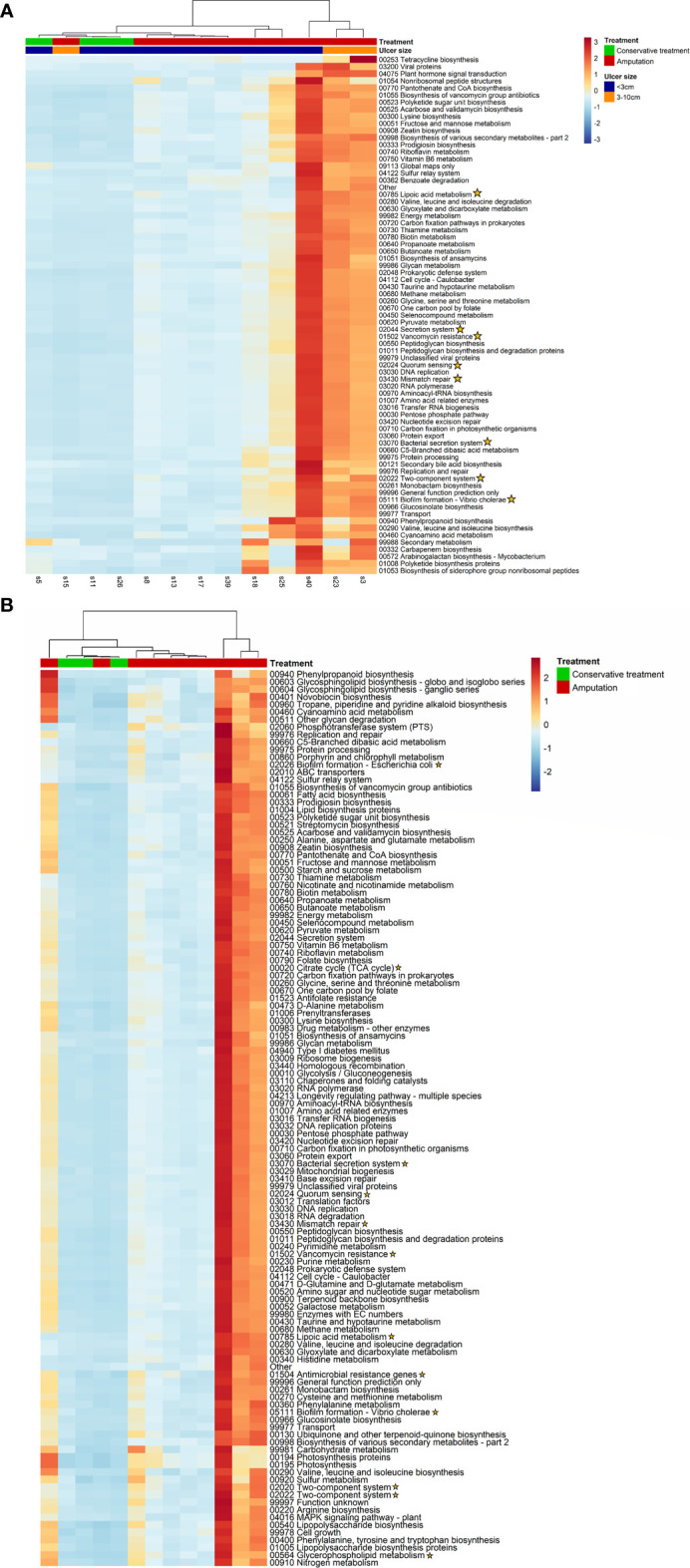
**(A, B)**. Analysis of functional genes. Yellow stars indicate genes related to bacterial virulence factors. **(A)** A cluster of five samples of patients with IDFU who underwent amputation. These patients had more severe peripheral vascular disease compared to other patients, p=0.003. **(B)** Functional genes differentiating patients who underwent amputation from patients who were treated conservatively.

Bacterial functional genes were sequenced and analyzed. A cluster of five samples of patients who underwent amputation was found; 220 pathway genes were more prevalent in these five IDFU compared to other samples. Among them were resistance genes for beta-lactam and vancomycin (p<0.001), Pseudomonas aeruginosa, Escherichia coli and Vibrio-cholera biofilm formation genes (p<0.001) and genes associated with bacterial virulence factors (**Figure 6A** and **Table S4A**). Moreover, these patients had more peripheral vascular disease compared to other patients (p=0.003), and their IDFU yielded more cultured bacteria with AMP-C β-lactamase resistant mechanism (p=0.017).

Furthermore, analysis of associations between each functional gene and clinical outcomes revealed genes that were more prevalent in patients who underwent amputation. These included, among others, resistance genes for vancomycin, genes related to biofilm production and other genes related to virulence factors (p<0.001, **Figure 6B** and **Table S4B**).

## Discussion

This study explored the bacterial bioburden of IDFU and its association with clinical outcomes, in hospitalized patients. We based our results on biopsies, which are considered more reliable than swab sampling (Pellizzer et al., 2001; Lipsky et al., 2012; Rhoads et al., 2012a; Lipsky et al., 2020) and used three investigative tools: cultures, 16S rRNA sequencing and metagenomic sequencing. Our main findings were fivefold:

### Anaerobes and GNB Dominate IDFU

IDFU are considered a diverse, polymicrobial ecosystem with very heterogenous bacterial communities (Smith et al., 2016; Hitam et al., 2019). The bacterial bioburden of all 31 IDFU was indeed varied; however, we found shared general characteristics. First, 56% of bacteria identified in 16S rRNA sequencing and 76% in metagenomics were anaerobes. Anaerobes are considered commensals, relatively nonvirulent bacteria in immunocompetent patients, predominantly seen in deeper and more chronic wounds (Charles et al., 2015). The dominance of anaerobes in DFU was previously demonstrated in studies using molecular tools (Dowd et al., 2008), presumably secondary to microvascular lower limb ischemia. Their dominance in sequenced data emphasized the disadvantage of culture-based results, as less sensitive to anaerobes, and highlights the importance of molecular methods to improve our understanding of the true microbiome of IDFU. Second, GNB were more common than Gram-positive in all three methods. This finding strengthens the emerging body of data regarding the high prevalence of GNB in DFU, mainly in warmer countries around Asia and Africa (Al Benwan et al., 2012; Dunyach-Remy et al., 2016). In a study conducted in Pakistan on 473 specimens, 76% of the IDFU cultured had high abundance of GNB (Miyan et al., 2017). Data regarding the principal involvement of anaerobic and GNB in IDFU, based on sequenced results, can improve antibiotics suitability, and challenge the current common practice of de-escalating empiric antibiotic treatment based on culture results only (Lipsky et al., 2012; Lipsky et al., 2020).

### The Association Between Specific Genera/Species and Clinical Outcomes

As expected, *Staphylococcus aureus* was the most common pathogen isolated by cultures, yet this well-known cause of skin, soft tissue, and bone infections was less dominant in sequencing results. *Staphylococcus aureus* is known to be a relatively virulent pathogen associated with adverse clinical outcomes of DFU (Citron et al., 2007; Kalan et al., 2019). Unexpectedly, in this cohort, it was associated with 63% of conservatively treated IDFU vs. 23% of patients who underwent amputation (p=0.048). Furthermore, and counterintuitively, *the Staphylococcus* genus was related to small ulcers according to 16S rRNA results (<3cm, p<0.001). We assume that this finding might be explained by the fact that as the wound deepens, the *Staphylococcus* genus is masked by anaerobes which thrive in anaerobic environments. This accords with a 16S rRNA sequencing study that showed that superficial and short-duration ulcers were associated with relatively higher abundance of *Staphylococcus* compared to deep DFU (Gardner et al., 2013). Additionally, we believe that patients with more severe and chronic wounds were exposed to antibiotics directed against *Staphylococcus aureus*; therefore, it was less dominant in these ulcers.

Metagenomic results suggested a possible link between specific genera, species, and clinical outcomes. *Bacteroides* genus (specifically *bacteroides fragilis* and *xylanisolvens* species) were more prevalent in patients who underwent amputation (p=0.001). *Bacteroides fragilis* was described as a relatively common pathogen in IDFU (El-Tahawy, 2000; Abdulrazak et al., 2005; Citron et al., 2007). However, its connection to adverse clinical outcomes is less recognized. On the other hand, *Proteus mirabilis, Pseudomonas aeruginosa, Streptococcus agalactiae* and *Escherichia coli* were more prevalent among conservatively treated patients. To the best of our knowledge, these correlations have not been described previously. Limited by a relatively small sample size, we can only note that these bacteria may influence clinical outcomes. Further studies are needed to establish the role of these bacteria in wound healing.

### Ulcer Size Might Influence the IDFU Microbiome

Although higher risk for amputation was previously described in patients with large ulcers (Oyibo et al., 2001), we did not find correlations between ulcer size and clinical outcomes. However, our results suggest that ulcer size might affect the IDFU ecosystem. *Staphylococcus*, *Prevotella* and *Bilophila* genera were associated with ulcers smaller than 3 cm as reflected by 16S rRNA sequencing, as well as 11 additional species according to metagenomics. We also found 19 species that were more prevalent in larger ulcers, noted in **Table S3**. Few studies investigated the impact of ulcer size on the ulcer microbiome. One stated that deeper ulcers contain higher levels of anaerobes and have more diverse microbiota (Gardner et al., 2013). These findings require additional investigation to solve a “chicken and egg” situation – does ulcer size influence the microbiome or do certain bacteria cause the IDFU to increase?

### Resistance Mechanisms and Bacterial Virulence Factor Genes Associated With Adverse Clinical Outcomes

Among the cultured bacteria, 43% exhibited at least one well-known resistance mechanism (MRSA, AMP-C beta-lactamase, Extended spectrum beta-lactamase [ESBL] and multi-drug resistant GNB). We found that bacteria with AMP-C beta-lactamase resistance mechanism were more prevalent in patients who underwent amputation (41% vs. 30%, p=0.041); however, all other resistance mechanisms were distributed similarly among all 31 patients, whether amputated or not. Traditionally, bacterial resistance profiling based on cultures, provide limited and mostly phenotypic data; metagenomic data provide insights into the pathogenicity of the bacteria by detecting functional genes, some related to antibiotic resistance mechanisms, biofilm formation and virulence factors.

In this study, the functional metagenomics data revealed genes related to beta-lactam and vancomycin resistance pathways. Other genes related to antimicrobial resistance were found and were more common in patients who underwent amputation, however with no relation to specific antibiotic agent. The widespread presence of resistant bacteria in DFU was noted (Saltoglu et al., 2018); however, only a handful of studies used metagenomic approaches to evaluate the IDFU resistome related to clinical outcomes (Kallstrom, 2014). Recent research efforts attempted to characterize this impact; a meta-transcriptomic study used RNA sequencing to identify 132 functional pathway genes in 16 IDFU samples; some of which were associated with infection severity (Zakrzewski et al., 2020). In our cohort, vancomycin resistance genes were more common in patients with wounds that required amputation (**Figure 6B**).

Additionally, genes associated with bacterial virulence factors other than resistance mechanisms were correlated with IDFU that resulted in amputation; some were associated with biofilm production, quorum sensing and toxin producing. Given the relatively limited sample size, additional research is needed to establish the implications of these genes.

### Peripheral Vascular Disease Might Influence the IDFU Microbiome

While searching for clusters with shared genes and possible impact on clinical outcomes, a cluster of five patients who underwent amputation was found. These patients demonstrated unique, common pathway genes. In comparison, they shared more genes associated with bacterial virulence factors such as beta-lactam and vancomycin resistance, and pseudomonas aeruginosa and vibrio-cholera biofilm-forming genes (p<0.001, **Figure 6A**). More of these patients had peripheral vascular disease compared to others and their IDFU cultures grew more AMP-C resistant bacteria. PVD was previously associated with the tendency to develop unhealing ulcers in diabetic patients (Lipsky et al., 2006; Lavery et al., 2017; Shin et al., 2017; Lu et al., 2021); however, data regarding the microbiome of ulcers in patients with PVD are lacking. The decreased blood flow characterizing patients with PVD promotes poor wound healing. This may give an advantage to specific bacteria, mostly anaerobes, which impact clinical outcomes.

This study had several limitations. First, metagenomic analysis of tissue samples is considerably challenging. Unlike 16S rRNA sequencing, metagenomic sequencing does not include a PCR amplification step. Therefore, all available genomic material is sequenced. As the tissue samples consist primarily of human DNA, the likelihood of sequencing microbial DNA is decreased. Therefore, we decided to include only high-quality samples that could reliably describe the bacterial profile of the ulcer; a decision that led to limited sample size in the metagenomic analysis. Larger scale investigations are needed to validate our results. Second, deep tissue biopsies could only be taken by skilled, attending orthopedic surgeons sometimes after antibiotic therapy was initiated. Since biopsies are more reliable compared to swab-based sampling, we chose to make this methodological compromise. Furthermore, recent studies showed that antibiotic therapy does not shift the microbiome in a way that affects its composition or outcomes (Kalan et al., 2018). Accordingly, we assume that since biopsies were taken a median of one day after initiation of antibiotic therapy, this did not have much influence on the sequenced bacterial DNA. Culture samples were obtained before antibiotic initiation. Finally, we did not compare IDFU samples to normal skin samples or to samples obtained from uninfected DFU as controls. Since we based our results on deep tissue biopsies, sampling healthy skin or uninfected ulcers did not seem justified.

## Conclusion

Molecular sequencing tools improve our knowledge of the complex microbial biodiversity of IDFU and emphasize the high prevalence of anaerobes and GNB in these ulcers. Sequencing results highlight possible associations among certain genera and species to clinical outcomes, such as associations between *Bacteroides* genus and amputation and between *Staphylococcus aureus* and smaller ulcers. Metagenomic analysis additionally reveals associations between functional genes related to bacterial virulence factors and amputation. Our study demonstrates the importance of investigating the microbiome of IDFU and its potential in improving treatment strategies.

## Data Availability Statement

The original contributions presented in the study are publicly available. This data can be found here: National Center for Biotechnology Information (NCBI) BioProject database under accession number PRJNA741238.

## Ethics Statement

The study was performed in accordance with the declaration of Helsinki and was approved by the Institutional Ethics Committee of Meir Medical Center (0143-18MMC). Patients provided signed informed consent. The patients/participants provided their written informed consent to participate in this study.

## Author Contributions

Conception and design of study: MC, HM-Z, NG-Z, SC, and MK. Acquisition of data: HM-Z, AZ, YC, and YP. Analysis and interpretation of data: MC, SC, HM-Z, NG-Z, and TG. Drafting the manuscript: HM-Z. Revising the manuscript critically for important intellectual content: MC, NG-Z, and SC. Approval of the version of the manuscript to be published: HM-Z, SC, TG, AZ, MK, YC, YP, NG-Z, and MC. MC, HM-Z, NG-Z, and SC are the guarantors of this work and, as such, had full access to all the data in the study and take responsibility for the integrity of the data and the accuracy of the data analysis.

## Funding

This work was supported by the Technion Institute of Technology, “Keren Hanasi”, Cathedra, the Technion Integrated Cancer Center, the Alon Fellowship for Outstanding Young Researchers, the Israeli Science Foundation (grant 1571/17), the Seerave Foundation, the German-Israeli Foundation for Scientific Research and Development (grant I-1076-416.6-20), the Canadian Institute for Advanced Research (Azrieli Global Scholars; grant FL-000969), the Israel Cancer Research Fund (grant 1016142), Human Frontier Science Program Career Development Award (grant CDA00025/2019-C), and the Gutwirth foundation award. NG-Z is a CIFAR Fellow in the Humans & the Microbiome Program and a Horev Fellow (Taub Foundation). The Applebaum family foundation #11916.

## Conflict of Interest

The authors declare that the research was conducted in the absence of any commercial or financial relationships that could be construed as a potential conflict of interest.

## Publisher’s Note

All claims expressed in this article are solely those of the authors and do not necessarily represent those of their affiliated organizations, or those of the publisher, the editors and the reviewers. Any product that may be evaluated in this article, or claim that may be made by its manufacturer, is not guaranteed or endorsed by the publisher.
