# Supplementary material for: Microbiome Characterization of Infected Diabetic Foot Ulcers in Association With Clinical Outcomes: Traditional Cultures Versus Molecular Sequencing Methods
Source: Front Cell Infect Microbiol. 2022 Mar 24;12:836699. doi: 10.3389/fcimb.2022.836699 (PMC8987016; doi:10.3389/fcimb.2022.836699)
Supplement: Supplementary file 1 [file DataSheet_1.pdf]

Supplemental Materials

Culturing techniques: Biopsies were cultured in the clinical microbiology laboratory at Meir Medical Center on 5% sheep blood, Columbia CNA, CDC anaerobic blood, chrom agar Staph. aureus/MRSA (HyLab, Rehovot, Israel), chocolate, MacConkey and CDC anaerobic Blood+gentamycin plates (NOVAmed, Jerusalem, Israel). Solid biopsies were additionally cultured in 2.5 ml BHI broth (HyLab, Rehovot, Israel), and then transferred to solid agar plates using standard methods [1]. After incubation at 35°C for 24hr, bacteria were identified using matrix-assisted laser desorption ionization time-of-flight (MALDI-TOF) mass spectrometry (Vitek MS, BioMerieux, Inc., Marcy l' Etoile, France) or Vitek-2 analysis (BioMérieux, Inc.). Antimicrobial susceptibility testing was determined using Vitek-2 analysis (BioMérieux, Inc.) and/or by disk diffusion assay (Oxoid, Basingstoke, UK) and/or by the gradient method (Etest®, Biomérieux, Inc.) according to procedures established by Clinical and Laboratory Standards Institute (CLSI) guidelines [2].

DNA extraction protocol: Five-thousand-two-hundred mg of tissue from each sample were defrosted on ice. Genomic DNA was extracted using DNeasy PowerBiofilm Kit (Qiagen, Hilden, Germany) according to the manufacturer's protocol. PCR, qPCR and microvolume spectrophotometer (NanoDrop) methods were used to determine the microbial DNA load in the sample to make sure it was suitable for sequence analysis. NEBNext Microbiome DNA Enrichment Kit (New-England Biolabs, USA) was used as part of a feasibility test to enrich the microbial DNA in one random sample, by selective binding and removal of the CpG-methylated host DNA. However, qPCR results and metagenomic sequencing analysis did not show a significant difference before and after enrichment. Therefore, we did not use this kit with other samples.

DNA sequencing: 16SrRNA and metagenomic sequencing were performed for each biopsy at the Sequencing Core at the University of Illinois at Chicago.

16SrRNA sequencing: Genomic DNA was PCR amplified with primers CS1_515F and CS2_806R (modified from the primer set employed by the Earth Microbiome Project (EMP; GTGYCAGCMGCCGCGGTAA and GGACTACNVGGGTWTCTAAT) targeting the V4 regions of microbial small subunit ribosomal RNA genes. Amplicons were generated using a two-stage PCR amplification protocol [3] and sequenced using the Illumina MiniSeq. 16SrRNA sequence data were processed with QIIME2 (version 2020-8), using built-in DADA2 software for sequence modelling and taxonomic assignment was performed against the SILVA reference database (release 132) [4,5].

High-throughput shotgun metagenomic sequencing: Genomic DNA samples were prepared for sequencing by an initial quantification using Qubit 4 Fluorometer (Life Technologies, #Q32851, Grand Island, NY). Library preparation was performed using the Nextera FLEX Workflow with UDI indexing (#20018705, 20027213 Illumina Inc San Diego, CA) according to the manufacturer's instructions with 5 ng template input and 9 cycles of PCR. An equal-volume pool of all libraries was then made. The pool was quantified using a Qubit DNA High Sensitivity kit (Life Technologies, #Q32851, Grand Island, NY), and size distribution was assessed using an Agilent 4200 TapeStation System (Agilent Technologies, G2991AA, Santa Clara, CA) using TapeStation D5000 ScreenTape, ladder and assay (Agilent Technologies, # 5067-5588, 5067-5590 and 5067-5589, Santa Clara, CA). The pooled libraries were run on Illumina MiniSeq instrument using MiniSeq Reagent MO Kit, (300 cycles) (Illumina Inc San Diego, CA) run for quality control and libraries balancing purposes. A new pool was made based on the MiniSeq run results, quantified same as described above

and sequenced on an Illumina NextSeq 500 instrument (300 cycles) (Illumina Inc San Diego, CA), with a 1% phiX spike-in.

Taxonomic profiling: Raw reads were mapped to the NCBI nucleotide database using Centrifuge [6]. Taxonomic annotations for each read were obtained using least common ancestor algorithm, and then summarized across all reads to create counts per taxon. Samples with less than 10,000 assigned reads were filtered. Raw counts were normalized using total sum scaling.

Functional profiling: Raw reads were mapped to the Swissprot protein database using DIAMOND [7,8]. Gene orthologs annotations were then assigned using the consensus of aligned references and then summarized across all reads to create counts per ortholog for each sample. Higher level summaries of orthologous functions were created using KEGG BRITE hierarchical annotations [9]. Raw counts were normalized to percentages for relative abundance.

<u>Statistics and bioinformatics</u>*:* Statistical analysis of Sequenced data was initially performed using MicrobiomeAnalyst [10] followed by comprehensive analysis using R packages: Phyloseq [11], Vegan [12] and DESeq2 [13]. Differences in microbial taxa and functional modules were assessed by differential abundance analyses using DESeq2 [13]. Beta diversity distance matrices (Bray-Curtis) were compared using the vegan package’s function ADONIS, a multivariate ANOVA based on dissimilarity tests and visualized using PCoA (taxonomy) and PCA (functional). Results were visualized using the ggplot2 R package [14].

<u>Raw sequenced data</u>:

16SrRNA sequencing: Mean number of reads was 29897 (SD+/-7985.398, max:57684, min:14879); 1 at the Kingdom level, 25 at the Phylum level, 37 at the Class level, 108 at the Order level, 183 at the Family level and 300 at the Genus

level.

High-throughput shotgun metagenomic sequencing: Mean number of reads was 230580.3 (SD+/-317222.4, max:1009260, min:16986); the dataset has 4531 taxa distributed into 1 at the Kingdom level, 45 at the Phylum level, 90 at the Class level, 184 at the Order level, 398 at the Family level, 1185 at the Genus level and 4026 at the Species level.

Tables

Table S1. Peripheral vascular assessments of patients included in the study

**Patient no.**	**Peripheral vascular diseases**	**ABI/TBI[a]**	**Ischemia grade[b]**
1	No	NA[c]	NA
2	Yes	0.68/0.46	1
3	No	NA	NA
4	No	NA	NA
5	No	NA	NA
6	Yes	Severe diseases a/t CTA[d]	3
7	Yes	0.94/0.46	1
8	Yes	0.86/0.58	1
9	Yes	0.88/0.46	1
10	No	NA	NA
11	No	NA	NA
12	Yes	No pulse/0.25	3
13	No	NA	NA
14	Yes	0.64/0.16	3
15	No	NA	NA
16	No	NA	NA
17	No	NA	NA
18	No	NA	NA
19	No	NA	NA
20	Yes	0.44/No pulse	3
21	No	NA	NA
22	No	NA	NA
23	Yes	1.2/0.25	3
24	No	NA	NA

25	No	NA	NA
26	No	NA	NA
27	Yes	1.05/0.21	3
28	No	NA	NA
29	No	NA	NA
30	Yes	No pulse/0.58	3
31	No	NA	NA

Assessments were performed during hospitalization or 6 months before admission/after discharge
a ABI=Ankle Brachial Index; TBI= Toe Brachial Index
b Ischemia grade: o=ABI≥0.8 (ankle systolic pressure>100mm Hg), 1=0.6<ABI<0.79 (ankle systolic pressure 70-100mm Hg), 2=0.41<ABI<0.59 ((ankle systolic pressure 50-70mm Hg),, 3=ABI≤0.39 (ankle systolic pressure<50mm Hg), TBI<0.7 was considered diagnostic for PVD, TBI<0.25 was considered as severe ischemia.
[c] NA – not applicable
[d] CTA=Computed tomography angiography

Table S2. Comparison between the three investigative tools

a. Traditional culture versus 16S rRNA sequencing results

**Bacterial classification characteristics**	**Traditional culture (N=30)**	**16SrRNA (N=30)**	**P-value**
Gram stain, mean (±SD)			
Percentage of gram-positive bacteria	48.8 (±28.5)	39.5 (±9.2)	0.07
Percentage of gram-negative bacteria			
Oxygen requirements, mean (±SD)			
Percentage of aerobic bacteria	19.9 (±23.4)	19.2 (±19.5)	1.00
Percentage of anaerobic bacteria	16.3 (±18.8)	56.2 (±18.5)	<0.001
Percentage of facultative anaerobic bacteria	63.8 (±26.7)	24.6 (±7.2)	<0.001

b. Traditional culture versus 16SrRNA sequencing versus metagenomic sequencing results

**Bacterial classification characteristics**	**Traditional culture (N=13)**	**16SrRNA (N=13)**	**Metagenomics (N=13)**	**P-value**
Gram stain, mean (±SD)				
Percentage of gram-positive bacteria	45.6 (±17.0)	41.1 (±8.6)	21.4 (±13.7)	0.001
Percentage of gram-negative bacteria				
Oxygen requirements, mean (±SD)				
Percentage of aerobic bacteria	15.4 (±15.8)	15.9 (±18.1)	5.0 (±2.9)	0.404
Percentage of anaerobic bacteria	25.9 (±21.7)	58.7 (±19.3)	76.3 (±11.9)	<0.001
Percentage of facultative anaerobic bacteria	58.7 (±26.1)	25.3 (±9.5)	17.8 (±12.4)	0.001

Table S3: Associations between specific species and ulcer size according to metagenomic sequencing results

A. Species that were more common in IDFU smaller than 3cm

Species	log2FoldChange	Adjusted P-value
Veillonella parvula	9.57	<0.001
Fusobacterium nucleatum	8.71	0.001
Veillonella rodentium	6.73	0.005
Streptococcus oralis	6.64	0.007
Veillonella atypica	6.03	0.013
Streptococcus sp. VT 162	5.90	0.01
Ornithobacterium inotracheale	3.59	0.03
Eikenella corrodens	10.03	0.04
Prevotella bivia	9.95	0.05
Prevotella denticola	3.64	0.05
Prevotella fusca	3.70	0.05

B. Species that were more common in IDFU 3-10cm

Species	log2FoldChange	Adjusted P-value
Moraxella osloensis	8.99	<0.001
Lachnoclostridium sp. YL32	10.37	<0.001
Bacteroides thetaiotaomicron	7.28	<0.001
Eggerthella lenta	8.32	<0.001
Dialister pneumosintes	8.05	<0.001
Clostridium bolteae	7.47	<0.001

Lactobacillus rhamnosus	6.24	<0.001
Campylobacter ureolyticus	4.73	<0.001
Peptoniphilus sp. ING2-D1G	3.83	<0.001
Parabacteroides distasonis	5.57	<0.001
Parabacteroides sp. CT06	4.57	<0.001
Intestinimonas butyriciproducens	4.40	<0.001
Pseudomonas aeruginosa	3.23	<0.001
Roseburia hominis	4.89	<0.001
Flavonifractor plautii	5.36	0.001
Bacteroides caccae	4.07	0.002
Proteus mirabilis	5.30	0.004
Mobiluncus curtisii	6.63	0.004
Bifidobacterium longum	3.02	0.006

Table S4. Metagenomic sequencing results: functional data analysis

A. Five patients cluster – all underwent amputation

Gene	Log2FC	LfcSE	P value	FDR
Vancomycin resistance	2.59	0.59	<0.001	<0.001
Beta-lactam resistance	2.41	0.37	<0.001	<0.001
Quorum sensing	2.71	0.45	<0.001	<0.001
Biofilm production by Escherichia coli	2.44	0.47	<0.001	<0.001
Biofilm formation - Pseudomonas aeruginosa	2.27	0.53	<0.001	<0.001
Biofilm formation - Vibrio cholerae	2.80	0.43	<0.001	<0.001
Bacterial secretion system	2.30	0.50	<0.001	<0.001
Mismatch repair	2.53	0.46	<0.001	<0.001
Citrate cycle (TCA cycle)	2.36	0.43	<0.001	<0.001
Lipoic acid metabolism	3.17	0.70	<0.001	<0.001
Two-component system	2.72	0.48	<0.001	<0.001
Glycerolipid metabolism	0.96	0.23	<0.001	<0.001
Bacterial toxins	1.24	0.49	0.006	0.008

B. Pathway genes associated with amputation

Gene	Log2FC	LfcSE	P-value	FDR
Vancomycin resistance	3.24	0.78	<0.001	<0.001
Quorum sensing	2.63	0.78	<0.001	0.008
biofilm production by Escherichia-coli	2.35	0.76	0.002	0.012

Biofilm formation - Pseudomonas aeruginosa	1.50	0.87	0.08	0.218
Biofilm formation - Vibrio cholerae	2.41	0.83	0.004	0.02
Bacterial secretion system	3.03	0.85	<0.001	0.007
Mismatch repair	2.53	0.75	<0.001	0.008
Citrate cycle (TCA cycle)	2.36	0.71	<0.001	0.008
Lipoic acid metabolism	3.24	1.02	0.002	0.01
Two-component system	2.03	0.70	0.003	0.018
Glycerolipid metabolism	0.90	0.36	0.012	0.052

Figures

Figure S1. Mean relative abundance of dominant bacteria in 16S rRNA [A] and metagenomic [B] sequencing results

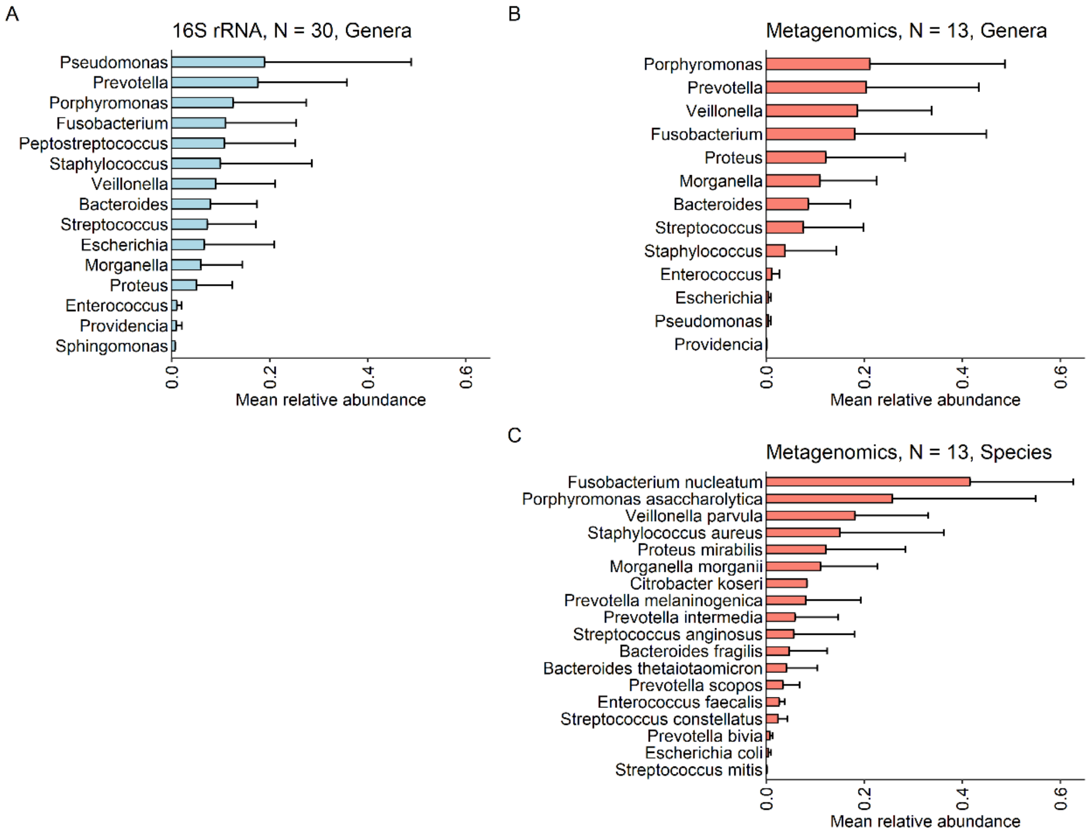

Figure S2. Associations between certain genera [A] and species [B] and amputation/conservative treatment, according to metagenomic sequencing analysis

A

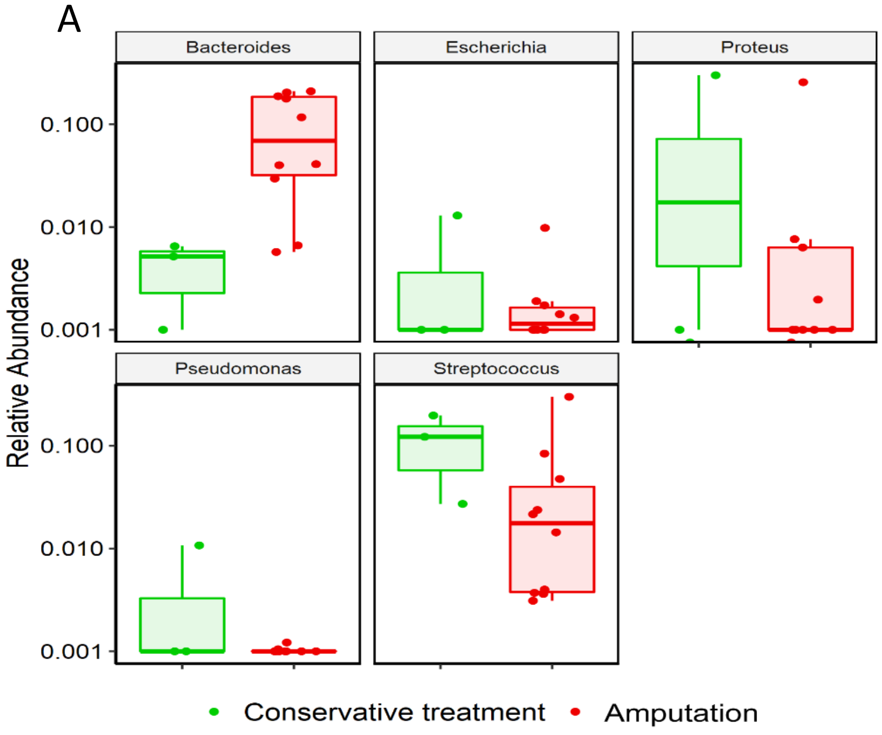

B

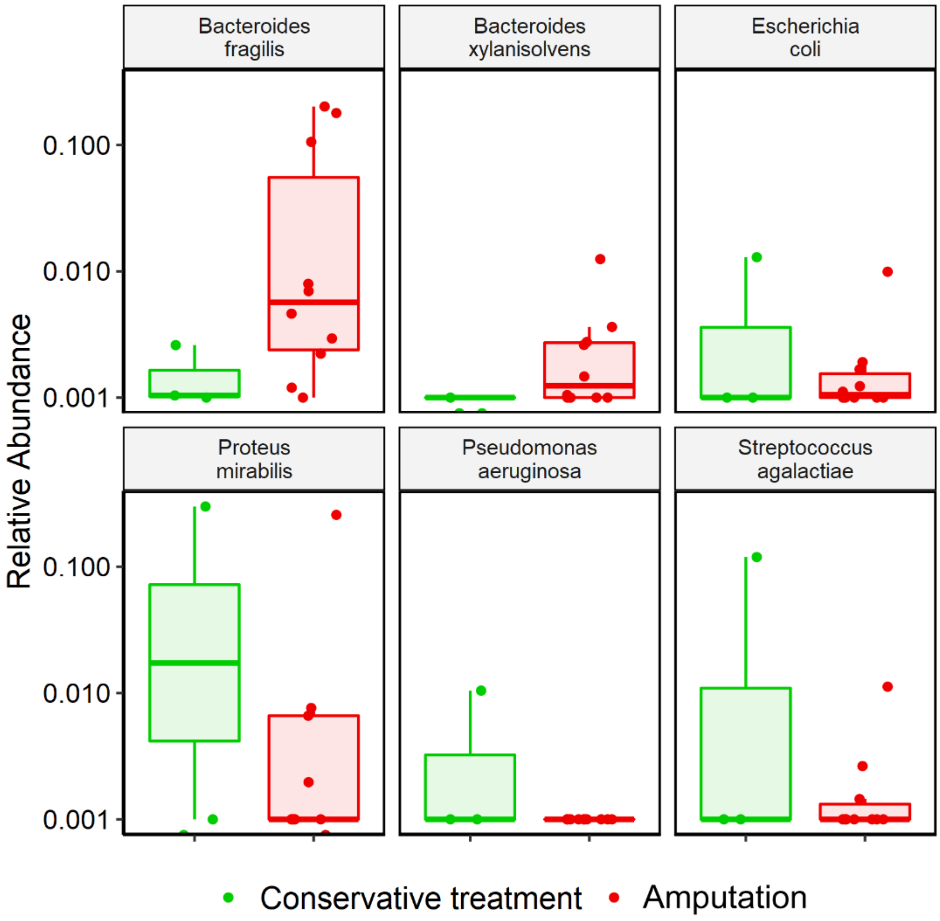

Figure S3. NMDS analysis of metagenomic results – Ulcer size

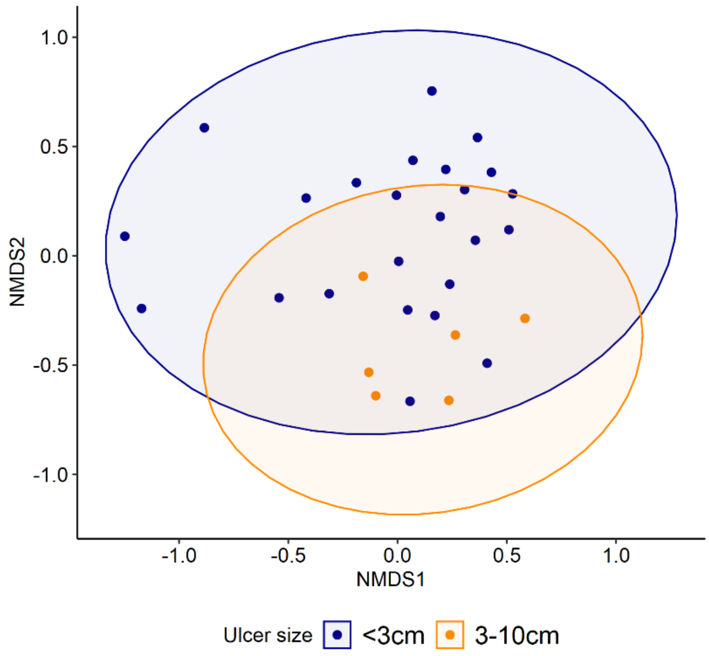

Figure S4. Associations between certain species and ulcer size according to metagenomic sequencing analysis

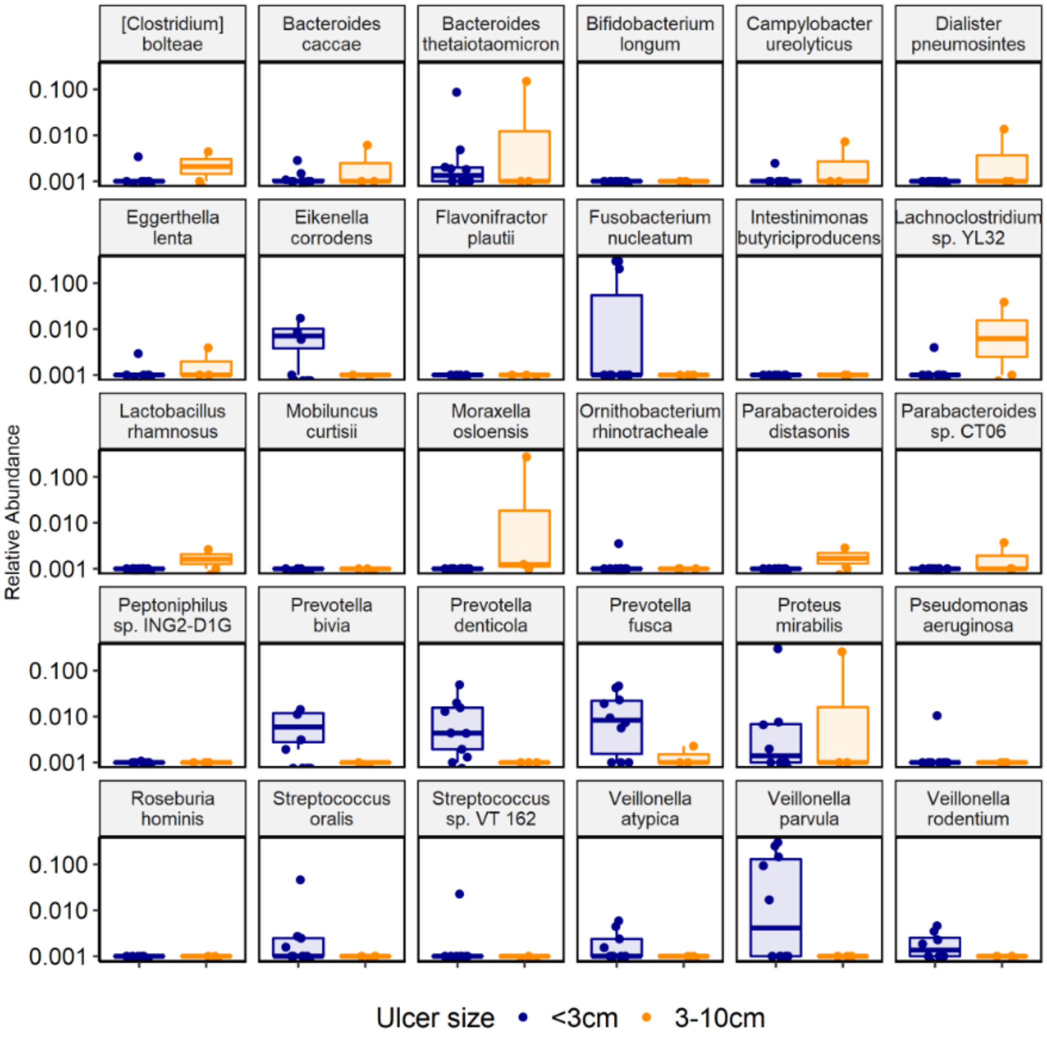

References

1. Murray PR, Baron ER, Pfaller MA, Tenover FC, Yolken RH. Manual of clinical microbiology. 6th ed. Washington DC: American Society for Microbiology; 1995
2. Weinstein MP, Patel JB, Lewis JS, Bobenchik AM, Campeau S, Cullen SK et al. Clinical and Laboratory Standards Institute (CLSI). Performance Standards for Antimicrobial Susceptibility Testing. 24th ed to 29th ed. Clinical and Laboratory Standards Institute: Wayne, PA, USA; 2016. Available from: https://clsi.org/media/3481/m100ed30_sample.pdf.
3. Poggi, S, Wang W, Hyde M, Kunstman K. Green, SJ. Making and sequencing heavily multiplexed, high-throughput 16S ribosomal RNA gene amplicon libraries using a flexible, two-stage PCR protocol. Methods Mol Biol 2018;1783:149-169. doi: 10.1007/978-1-4939-7834-2_7.
4. Bolyen E, Rideout JR, Dillon MR et al. Reproducible, interactive, scalable and extensive microbiome data science using QIIME 2. Nat Biotechnol 2019;37:8:852-7. doi: 10.1038/s41587-019-0209-9.
5. Callahan BJ, McMurdie PJ, Rosen MJ, Han AW, Johnson AJA, Holmes SP. DADA2; high-resolution sample inference Illumina amplicon data. Nat Methods 2016;13:7:581-3. doi:10.1038%2Fnmeth.3869
6. Kim D, Song L, Breitwieser FP, Salzberg SL. Centrifuge: rapid and sensitive classification of metagenomic sequences. Genome Res 2016;26:12:1721-1729. doi: 10.1101/gr.210641.116.
7. Buchfink B, Xie C, Huson DH. Fast and sensitive protein alignment using DIAMOND. Nature methods 2015;12:1:59-60. doi:10.1038/nmeth.3176.

8. Bateman A, Martin MJ, O'Donovan C et al. UniProt: the universal protein knowledge base. Nucleic Acids Res 2017;45:D1:D15-D169. doi:10.1093/nar/gkw1099
9. Kanehisa M, Furumichi M, Tanabe M, Sato Y, Morishima K. KEGG: new perspectives on genomes, pathways, diseases and drugs. Nucleic Acids Res 2017;45:D1:D35-D361. doi:10.1093/nar/gkw1092.
10. Dhariwal A, Chong J, Habib S, King IL, Agellon LB, Xia J. MicrobiomeAnalyst: A web-based tool for comprehensive statistical, visual and meta-analysis of microbiome data. Nucleic Acids Res 2017;45:W1:W180-W188. doi:10.1093/nar/gkx295.
11. McMurdie PJ, Holmes S. Shiny-phyloseq: Web application for interactive microbiome analysis with provenance tracking. Bioinformatics 2015;31:2:282-283. doi:10.1093/bioinformatics/btu616.
12. Oksanen J, Blanchet FG, Friendly M, Roeland K, Pierre L, Dan M et al. vegan: Community Ecology Package: Community Ecology Package. R package version 2.5-6. 2020. Available from: https://cran.r-project.org/package=vegan.
13. Love MI, Huber W, Anders S. Moderated estimation of fold change and dispersion for RNA-seq data with DESeq2. Genome Biology 2014;15:550. doi: 10.1186/s13059-014-0550-8.
14. Wickham H. ggplot2: Elegant Graphics for Data Analysis. Springer-Verlag New York 2016. Available from: https://www.springer.com/gp/book/9783319242750.
